# Highly efficient red-emitting Ca_2_YSbO_6_:Eu^3+^ double perovskite phosphors for warm WLEDs†

**DOI:** 10.1039/c9ra03410b

**Published:** 2019-07-03

**Authors:** Meijiao Liu, Biao Shen, Keyuan Wang, Jiasong Zhong, Daqin Chen

**Affiliations:** Department of Chemistry, Zhejiang Sci-Tech University Hangzhou 310018 China; College of Materials and Environmental Engineering, Hangzhou Dianzi University Hangzhou 310018 China jiasongzhong@hdu.edu.cn; College of Physics and Energy, Fujian Normal University Fuzhou 350117 P. R. China dqchen@fjnu.edu.cn

## Abstract

Highly efficient red-emitting Eu^3+^-doped double perovskite Ca_2_YSbO_6_ phosphors have been successfully prepared by the traditional high-temperature solid state method. The phase purity, photoluminescence and decay properties as a function of the Eu^3+^ concentration have been investigated in detail. The XRD results demonstrate that all of the obtained phosphors can be assigned to a pure monoclinic structure. Upon 464 nm excitation, a strong red emission situated at 614 nm (^5^D_0_–^7^F_2_) indicates that Eu^3+^ ions occupy a site with low symmetry. The quenching concentration of Eu^3+^ ions reaches as high as 70 mol% and the quenching mechanism is discussed. Especially, the prepared phosphor exhibits a high quantum efficiency of 92.1% and superior thermal stability, with PL intensity at 423 K up to 81.6% of that at room temperature. Moreover, a warm white light with a correlated color temperature of 4720 K and a color rendering index of 82.6 is achieved by fabricating a Ca_2_YSbO_6_:Eu^3+^ phosphor in a 460 nm blue-InGaN chip together with the commercial Y_3_Al_5_O_12_:Ce^3+^ yellow phosphor.

## Abstract 2

A double perovskite Ca_2_YSbO_6_:Eu^3+^ red-emitting phosphor with high concentration quenching and excellent quantum efficiency was developed to find a potential application in warm WLEDs.

## Graphical abstract

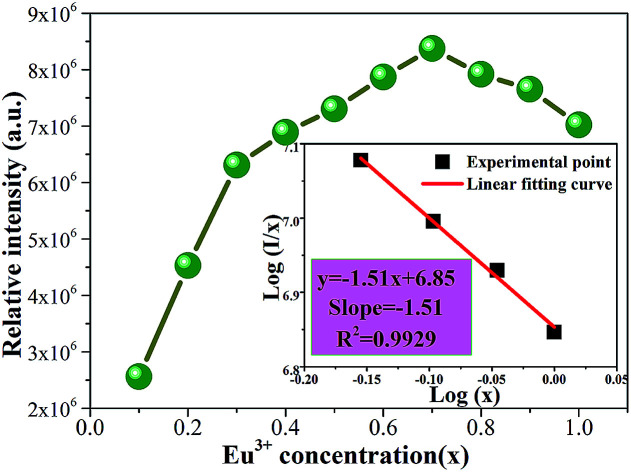

## 1. Introduction

In the past couple of decades, white light-emitting diodes (WLEDs), as the fourth generation of illumination appliances, have received significant interest due to their superior characteristics such as low electricity consumption, long operating lifetime, high brightness, good reliability, fast response, environmental friendliness, *etc.*^1–4^ Currently, the mainstream commercial phosphor-converted WLEDs (pc-WLEDs) are realized by combining a blue InGaN LED chip with a yellow YAG:Ce^3+^ phosphor.^5,6^ Although this combination has some advantages, this kind of white light demonstrates a low color rending index (CRI) and high correlated color temperature (CTT) owing to the deficiency of red light in the emission spectra.^7^ Therefore, in order to improve the CRI and CCT of pc-WLEDs, much effort has been devoted to develop a novel red phosphor.^8,9^ However, the most widely used rare earth ion activated red phosphors such as Y_2_O_3_:Eu^3+^, Y_2_O_2_S:Eu^3+^ and CaAlSiN_3_:Eu^2+^ also exhibit several limitations including low efficiency, chemical instability and complicated preparation processes, which prevent their broad application for lighting.^10–12^ Thus, it is urgent to search for a novel red phosphor with excellent stability, high conversion efficiency and low concentration quenching, which can be synthesized easily.

As the most important factor to acquire efficient red-emitting phosphors, different host lattices will demonstrate diverse optical properties due to the surrounding of the given optical center.^13^ It is well known that Eu^3+^ has generally been used as a luminescent center, however, the high efficiency red emission is mainly to be blamed for the low quenching concentration and improper coordination environment.^14^ Therefore, it is essential to find a suitable host matrix, in which the crystal structure benefits an increase in distance among the Eu^3+^ ions.^15^ Recently, ordered double perovskite type oxides with the general formula A_2_BB′O_6_ (A = Ca, Sr, Ba, Y, La, Gd; B = Y, La, Gd, Mg, Zn, Li; B′ = Sb, Ta, Nb, Ti) or AA′BB′O_6_ (A = Ca, Sr, Ba, Na, Li; A′ = Y, La, Gd; B = Mg, Zn; B′ = W, Mo, Sb, Ta, Nb) have been intensively investigated owing to their good thermal stability and chemical properties.^10,16^ In typical crystal structures of double perovskites, both the B and B′ sites are coordinated with six oxygen atoms by corner-sharing alternately, while the A (or A′) site is coordinated with eight or twelve oxygen atoms based on the distortion of the crystal structure. Besides, the BO_6_ and B′O_6_ sites can reduce the symmetry of the A (or A′) ones and produce various coordination environments for doping with rare earth ions.^17,18^ Therefore, the double perovskite oxide is a suitable candidate matrix for phosphors by structurally modulating.

Hence, we chose Ca_2_YSbO_6_ as the host for a red phosphor, in which the B and B′ sites are occupied by Y^3+^ and Sb^5+^ ions, respectively. In addition, Ca_2_YSbO_6_ belongs to an ordered perovskite structure and has a monoclinic crystal system with the space group of *P*2_1_/*m*, which is very suitable for luminescent ions.^19^ A Y^3+^ ion and six O^2−^ ions are coordinated to form [YO_6_] octahedrons, providing the opportunity for an Eu^3+^ ion to occupy the Y^3+^ lattice according to its similar ionic radius and the same valence. Recently, Bi^3+^ and Eu^3+^ ion co-doped Ca_2_YSbO_6_ phosphor materials have been synthesized, and the luminescence enhancement mechanism was also investigated.^10^ However, the influences of structure symmetry on high concentration quenching, thermal stability and quantum efficiency of Ca_2_YSbO_6_:Eu^3+^ red phosphors have not been studied in detail. Besides, the potential application for WLEDs is also unconsidered. Therefore, in this study, a series of Ca_2_Y_1−*x*_Eu_*x*_SbO_6_ (0.1 ≤ *x* ≤ 1.0) red-emitting phosphors have been prepared by the conventional high temperature solid-state reaction. In addition, the relationship between crystal structure and optical performance is also investigated and discussed in detail.

## 2. Experimental section

### 2.1 Preparation samples

A series of Ca_2_Y_1−*x*_SbO_6_:*x*Eu^3+^ (*x* = 0.1, 0.2, 0.3, 0.4, 0.5, 0.6, 0.7, 0.8, 0.9, and 1.0) phosphors were synthesized by the high-temperature solid state reaction method. Raw materials of CaCO_3_ (99.9%), Y_2_O_3_ (99.99%), Sb_2_O_5_ (99.99%) and Eu_2_O_3_ (99.99%) were weighed according to the stoichiometric ratio and ground thoroughly in an agate mortar for 30 min. After that, the mixtures were put into alumina crucibles and sintered at 1400 °C for 4 h. Subsequently, the final products were cooled to room temperature and ground again for further characterization.

### 2.2 Characterization

The crystal structure and phase purity of the final products were recorded on a MiniFlex 600 X-ray diffractometer with CuKα radiation (*λ* = 1.5405 Å) at a scanning rate of 1° min^−1^ in the 2*θ* range from 10° to 80°. The crystal structure parameters were refined by the Rietveld method using the General Structure Analysis System (GSAS). The morphology of the as-obtained product was studied using an FEI Apreo HiVac scanning electron microscope (SEM). The photoluminescence excitation (PLE) and emission (PL) spectra as well as luminescence decay lifetimes of the prepared phosphors were performed on an Edinburgh FS5 spectrometer using a 150 W continuous and pulsed xenon lamp as the excitation source, respectively. The internal quantum efficiency (IQE) of the synthesized phosphors was recorded on the Edinburgh FS5 spectrometer using an integrating sphere coated with BaSO_4_. The temperature dependent emission spectra were measured using a home-made temperature control system in the temperature range of 303–523 K.

### 2.3 Fabrication of WLEDs

The WLED devices were fabricated with the commercial YAG:Ce^3+^ yellow phosphor (purchased from XinLi Illuminant Co. LTD) and blue GaN chips (∼460 nm) with/without the as-prepared Ca_2_YSbO_6_:Eu^3+^ red phosphor. The mass ratio of yellow phosphor *versus* red one was 1 : 3. These phosphors were mixed with epoxy resin thoroughly and coated on the surface of the LED chips. The photoelectric parameters of the assembled WLEDs, including electroluminescence (EL) spectra, color rendering index (CRI), correlated color temperature (CCT), and luminescent efficiency (LE), were collected using an integrating sphere equipped with a CCD detector (HAAS-2000).

## 3. Results and discussion

### 3.1 Phase characterization and morphology analysis

**Fig. 1:**
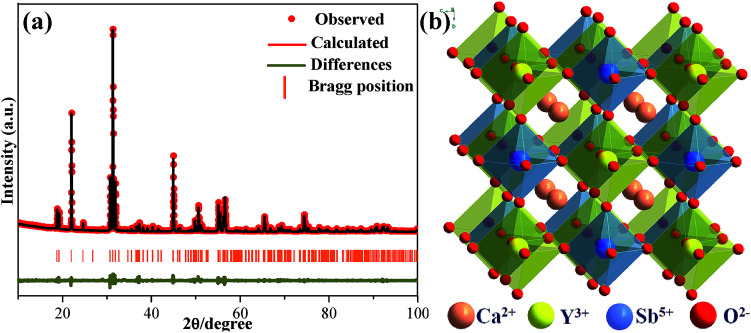
(a) Rietveld refinement XRD pattern of Ca_2_YSbO_6_:0.1Eu^3+^ sample. (b) Crystal structure of Ca_2_YSbO_6_.

The representative Ca_2_YSbO_6_:0.1Eu^3+^ sample was measured using powder X-ray diffraction (XRD), and the related data were analyzed using the Rietveld refinement method, as illustrated in Fig. 1a. Owing to the absence of structural information on Ca_2_YSbO_6_, we used the crystallographic data of Ca_3_TeO_6_ (ICSD #230130) as the initial structural model.^10^ The final refinement results of Ca_2_YSbO_6_:Eu^3+^ are listed in Table S1,† which index to a monoclinic structure with the lattice parameters *a* = 5.611 Å, *b* = 5.806 Å, *c* = 8.057 Å, and *V* = 262.48 Å^3^. The enlargement in cell volume certifies that the smaller Y^3+^ (*r* = 1.019 Å, CN = 8) lattice has been successful replaced by the larger Eu^3+^ (*r* = 1.066 Å, CN = 8) ions in the Ca_2_YSbO_6_. The schematic illustration of the Ca_2_YSbO_6_ structure is displayed in Fig. 1b, in which the Y^3+^ and Sb^5+^ ions form an alternative arrangement of [YO_6_] and [SbO_6_] octahedrons by sharing an O atom. Especially, the abundant [YO_6_] provides a better opportunity for Eu^3+^ ions to occupy the Y^3+^ lattice.

**Fig. 2:**
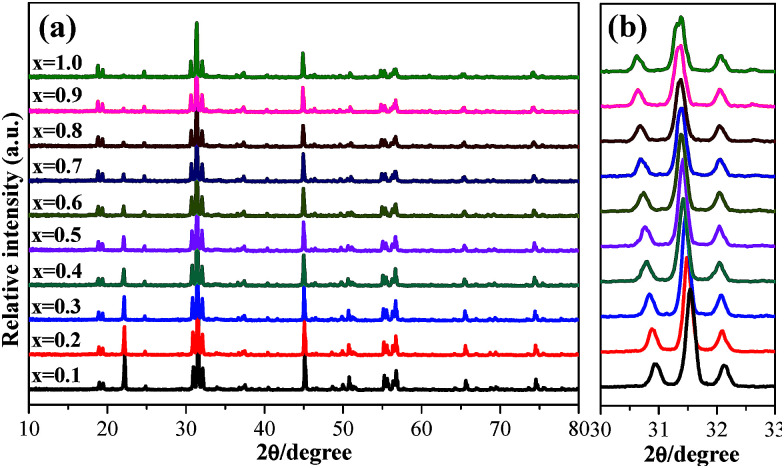
(a) XRD patterns of Ca_2_YSbO_6_:*x*Eu^3+^ with various Eu^3+^ content. (b) Partial enlarged diffraction peaks in the region between 30° and 33°.

The phase structures of the as-obtained Ca_2_YSbO_6_:Eu^3+^ phosphors with various Eu^3+^ concentrations were verified by XRD, as demonstrated in Fig. 2a. All the diffraction peak positions and relative intensities of the products appear at the same angles. No impurity and other phases can be found even with a doping concentration of 100 mol%, indicating that the final products have been successfully prepared and the doping with Eu^3+^ ions does not affect the crystal structure of the Ca_2_YSbO_6_ lattice. Magnification of the dominant diffraction peaks ranging from 30–33° is presented in Fig. 2b. It can be clearly seen that these peaks shift slightly to a lower angle value with the increase in Eu^3+^ concentration. This phenomenon can be explained according to Bragg’s equation, 2*d* sin *θ* = *nλ*, in which *θ* represents diffraction angle, *d* corresponds to interplanar distance, and *n* and *λ* are constants. Based on the effective ionic radii and the same valence state, Eu^3+^ (*r* = 1.066 Å, CN = 8) ions should substitute the Y^3+^ (*r* = 1.019 Å, CN = 8) lattice. Thus, the peak shifting is attributed to the replacement of the smaller Y^3+^ ions by larger Eu^3+^ ones. This result further indicates that Eu^3+^ ions have successfully entered into the Ca_2_YSbO_6_ lattice.

The surface morphology of the obtained Ca_2_Y_0.3_SbO_6_:0.7Eu^3+^ sample was measured using SEM analysis, as illustrated in Fig. S1.† Regular phosphor particles with the size range from 0.5 to 2.5 μm can be observed.

### 3.2 Luminescence properties

**Fig. 3:**
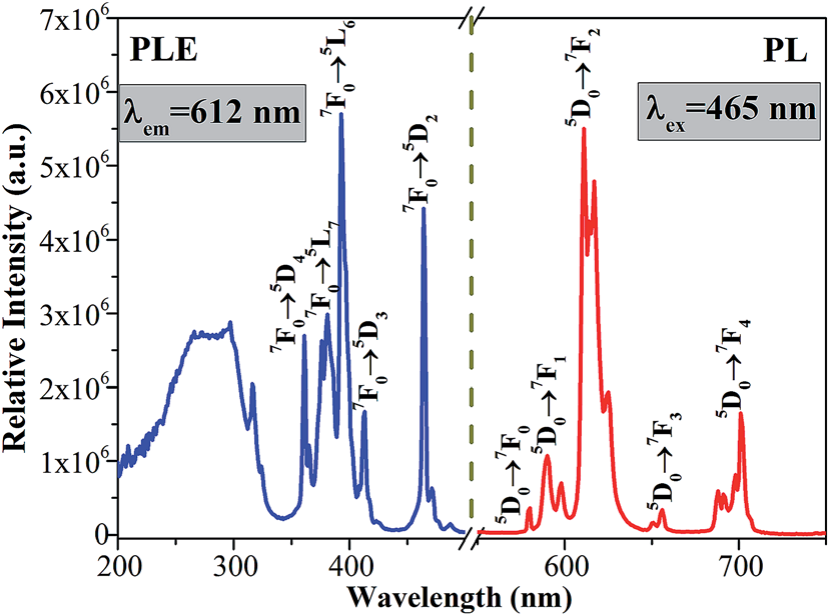
PLE and PL spectra of Ca_2_Y_0.3_SbO_6_:0.7Eu^3+^.

The PLE and PL spectra of the as-synthesized Ca_2_Y_0.3_SbO_6_:0.7Eu^3+^ powders are given in Fig. 3. When monitored at 612 nm, the PLE spectrum consists of several sharp peaks in the range of 350–500 nm and a broad band ranging from 200–350 nm, which are ascribed to the Eu^3+^ ions’ characteristic inner-4f transitions and the charge transfer band (CTB) of the O^2−^–Eu^3+^ interaction, respectively. The sharp peaks located at 316 nm, 361 nm, 380 nm, 393 nm, 413 nm and 464 nm are mainly attributed to the ^7^F_0_ → ^5^H_5_, ^7^F_0_ → ^5^D_4_, ^7^F_0_ → ^5^L_7_, ^7^F_0_ → ^5^L_6_, ^7^F_0_ → ^5^D_3_ and ^7^F_0_ → ^5^D_2_ transitions of the Eu^3+^ ion, respectively. Among them, two of the strongest sharp peaks at 393 nm and 464 nm can be found, which are in good agreement with the emission wavelength of NUV and blue LED chips.^20^ Upon 464 nm excitation, the characteristic emission peaks of Eu^3+^ ions corresponding to the ^5^D_0_ → ^7^F_*J*_ (*J* = 0, 1, 2, 3, 4) transitions are clearly observed. Especially, the most intense emission peak situated at 612 nm results from the electric dipole transition ^5^D_0_ → ^7^F_2_, which demonstrates that Eu^3+^ occupies a site with low symmetry in the double perovskite Ca_2_YSbO_6_ lattice.^21,22^ This non-inversion center environment of Eu^3+^ ions is beneficial to acquire a red phosphor with high color purity.^23^

**Fig. 4:**
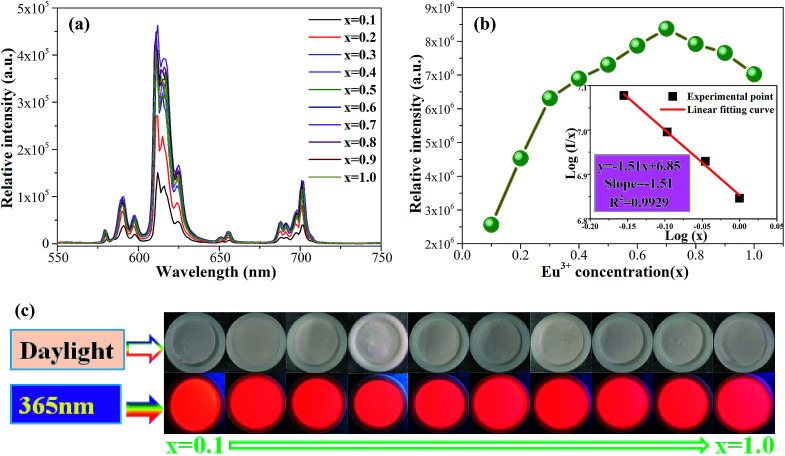
(a) Emission spectra of Ca_2_Y_1−*x*_SbO_6_:*x*Eu^3+^ (0.1 ≤ *x* ≤ 1.0) samples with different Eu^3+^ content excited at 464 nm. (b) The emission intensity of ^5^D_0_ → ^7^F_2_ as a function of Eu^3+^ concentration. Inset is the relation of log(*x*) *versus* log(*I*/*x*). (c) The photographs of corresponding samples under daylight and UV light.

In order to study the effect of Eu^3+^ concentration on the luminescence, the PL spectra of Ca_2_Y_1−*x*_SbO_6_:*x*Eu^3+^ (0.1 ≤ *x* ≤ 1.0) samples under the excitation of 464 nm are collected and presented in Fig. 4a. All of the samples display similar spectral profiles with the dominant red emission band located at 612 nm. As expected, the emission intensity was enhanced with the increase in Eu^3+^ doping concentration and reaches maximum at *x* = 70 mol%, as depicted in Fig. 4b. Then, the strength gradually decreases with further increasing Eu^3+^ concentration due to the well-known concentration quenching effect, which is ascribed to the probability of the energy migration between Eu^3+^–Eu^3+^ increasing. Herein, the high quenching concentration (70 mol%) in the Eu^3+^ doped Ca_2_YSbO_6_ double perovskite mainly originates from the different energy migrations between intralayer and interlayer luminescence centers.^21,24^ In the structure of Ca_2_YSbO_6_, [YO_6_] and [SbO_6_] are connected by corner-sharing alternately with a layered structure, which is beneficial to damp the concentration quenching among Eu^3+^–Eu^3+^ ions. Thus, higher emission intensity and luminescence efficiency can be realized *via* doping with a higher Eu^3+^ concentration.^15^ Besides, the critical distance (*R*_c_) among Eu^3+^ ions for energy transfer is an important parameter, which can be evaluated based on the Blasse theory.^25^ In this study, the *R*_c_ is estimated to be ∼7.1 Å, which is larger than 5 Å. Therefore, the concentration quenching is mainly governed by multipolar interactions. Herein, the type of interaction mechanism of Eu^3+^ ions in Ca_2_YSbO_6_ is determined by the relationship between log(*I*/*x*) and log(*x*) using the following expression:^26^1
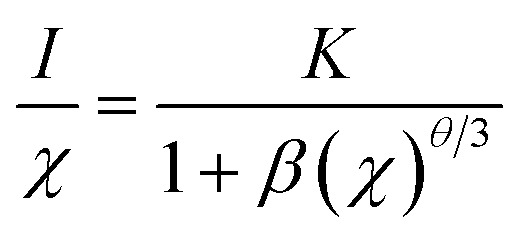
where *I* represents the emission intensity; *x* stands for the dopant concentration; *K* and *β* are constants for the given host; *θ* = 6, 8, and 10 correspond to dipole–dipole (d–d), dipole–quadrupole (d–q), and quadrupole–quadrupole (q–q) interactions, respectively. As illustrated in the inset of Fig. 4b, the relationship between log(*x*) and log(*I*/*x*) is found to be linear, and the slope is fitted to be about −1.51. Therefore, *θ* is calculated to be 4.53, which is close to 6, suggesting that the concentration quenching mechanism among Eu^3+^ ions in the Ca_2_YSbO_6_ is d–d interaction.

**Fig. 5:**
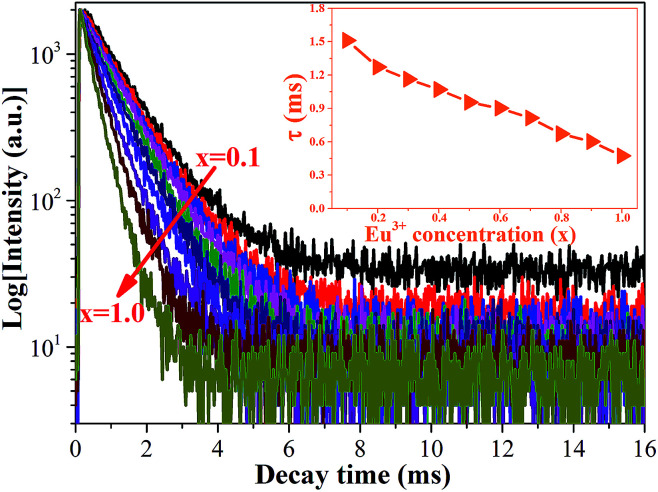
PL decay curves of Ca_2_YSbO_6_:*x*Eu^3+^ samples with different Eu^3+^ content. Inset is the average lifetime *versus* Eu^3+^ concentration.

In order to obtain additional information on the concentration quenching behavior of the Eu^3+^ ions, the luminescence decay curves of Ca_2_YSbO_6_:Eu^3+^ with various Eu^3+^ concentrations under the excitation of 464 nm are investigated, as illustrated in Fig. 5. All the decay curves of Eu^3+^ emission can be well fitted with a first order exponential as follows:^27^

**Fig. 6:**
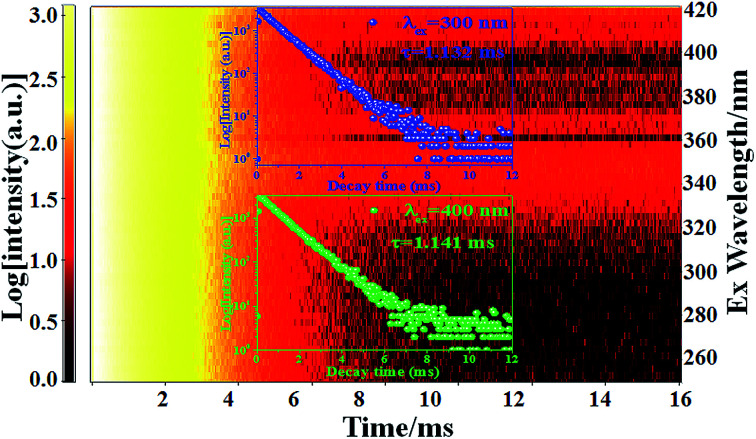
2D decay curves of Ca_2_Y_0.8_SbO_6_:0.2Eu^3+^ phosphor under various excitations ranging from 250–420 nm. Insets are the decay curves for excitation at 300 nm and 400 nm.

2 *I*(*t*) = *I*_0_ + *A* exp(−*t*/*τ*)

where *I*(*t*) and *I*_0_ correspond to the luminescence intensities at times *t* and 0, *A* is a constant, and *τ* is the decay time for the exponential components. The obtained average lifetimes monotonically decrease from 1.513 ms to 0.4733 ms with increasing Eu^3+^ concentrations from *x* = 0.1 to *x* = 1.0, which is mainly attributed to the enhanced non-radiative energy transfer among Eu^3+^ ions with shortening the distance between Eu^3+^–Eu^3+^. Besides, in order to further certify the emitting center in the Ca_2_YSbO_6_ lattice, the luminescence decay curves of Ca_2_Y_0.8_SbO_6_:0.2Eu^3+^ excited from 250–420 nm and monitored at 612 nm were measured, as depicted in Fig. 6. It can be clearly seen that the profile of all these curves is similar and the calculated *τ* values are almost the same. This result further confirms that the Eu^3+^ environment in this system is unique.

**Fig. 7:**
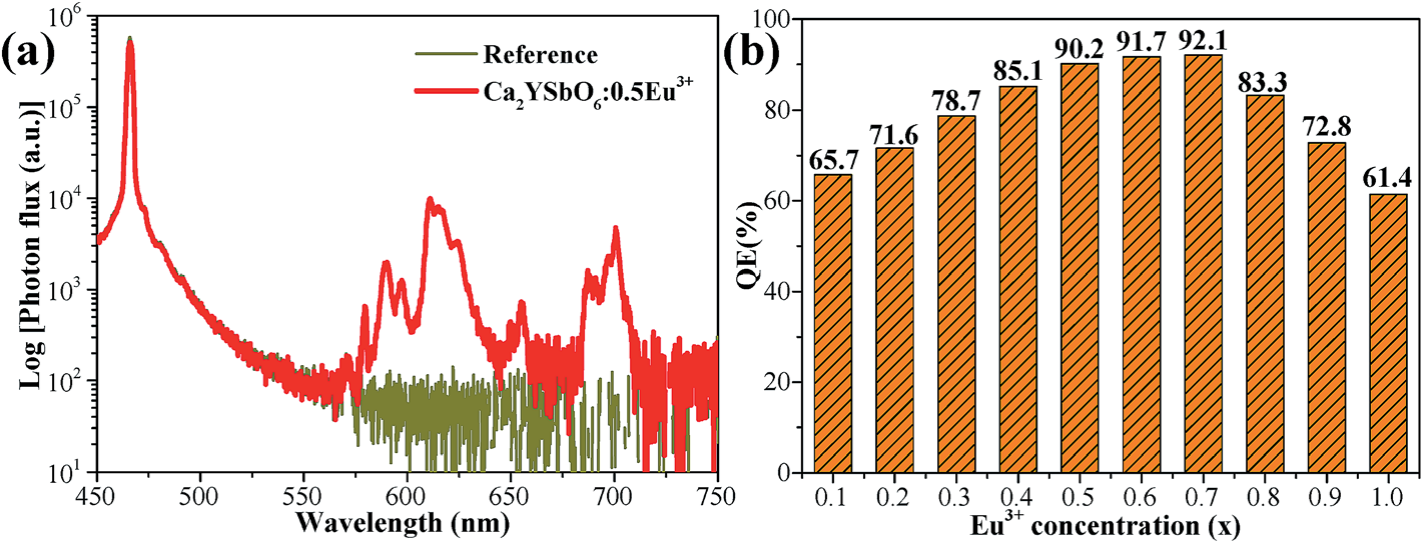
(a) Excitation and emission spectra of the Ca_2_Y_0.5_SbO_6_:0.5Eu^3+^ and reference sample recorded using an integrating sphere. (b) The IQE of Ca_2_Y_1−*x*_SbO_6_:*x*Eu^3+^ (0.1 ≤ *x* ≤ 1.0) products with various Eu^3+^ concentrations.

To evaluate the luminescence performance of the as-obtained phosphor materials for practical application, the internal quantum efficiency (IQE) was recorded. The measured spectrum of the Ca_2_Y_0.5_SbO_6_:0.5Eu^3+^ phosphor as an example and reference sample is given in Fig. 7, and the value can be estimated using the formula^28^3
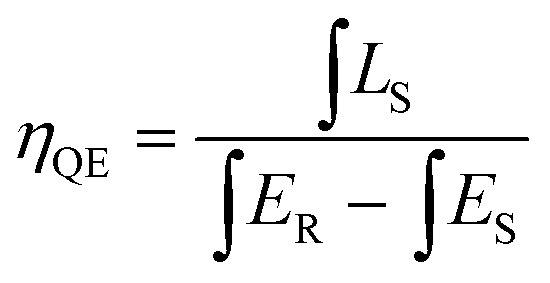
where *L*_S_ is the luminescence spectrum; *E*_R_ and *E*_S_ represent the spectra with and without Ca_2_Y_0.5_SbO_6_:0.5Eu^3+^ phosphor, respectively. Upon 464 nm excitation, the IQE values of Ca_2_Y_1−*x*_SbO_6_:*x*Eu^3+^ are decided to be 65.7%, 71.6%, 78.7%, 85.1%, 90.2%, 91.7%, 92.1%, 83.3%, 72.8% and 61.4% for *x* = 0.1, 0.2, 0.3, 0.4, 0.5, 0.6, 0.7, 0.8, 0.9 and 1.0, respectively. The variation tendency of IQE is in agreement with the concentration-dependent PL spectra discussed above, which is further evidence for the transformation of energy transfer caused by non-radiation. Especially, it is worth emphasizing that the IQE of the as-obtained phosphors is much higher than that of commercial phosphor Y_2_O_2_S:Eu^3+^ (IQE: 35%),^29^ illustrating that it has promising application prospects in solid-state lighting.

**Fig. 8:**
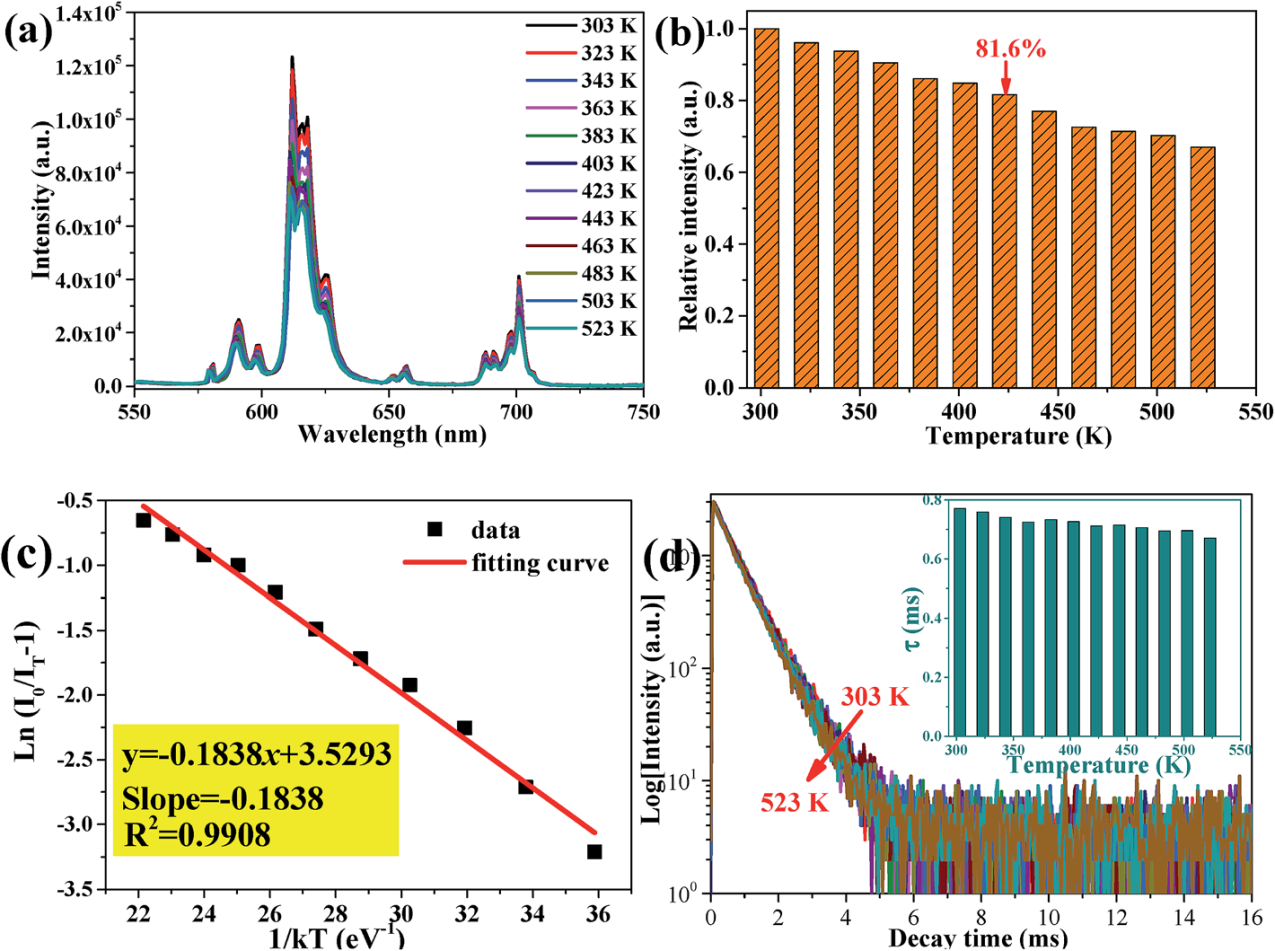
(a) Temperature-dependent emission spectra of Ca_2_Y_0.3_Eu_0.7_SbO_6_ phosphor ranging from 303–523 K. (b) Normalized PL emission intensity of Ca_2_Y_0.3_Eu_0.7_SbO_6_ sample as a function of temperature. (c) Plot of ln(*I*_0_/*I*_T_ − 1) *versus* 1/*kT*. (d) Temperature-dependent decay curves of Ca_2_Y_0.3_Eu_0.7_SbO_6_ sample excited at 464 nm. Inset is the lifetime under different temperatures.

As an indispensable performance objective in evaluating practical applications in solid-state lighting, the temperature quenching property has been investigated. The temperature-dependent PL emission spectra of the Ca_2_Y_0.3_Eu_0.7_SbO_6_ sample recorded from 303 to 523 K are illustrated in Fig. 8a. Unsurprisingly, as the temperature rises from 303 to 523 K, the emission peak remained almost unchanged except the PL emission intensity decreased gradually. Especially, the as-prepared Ca_2_YSbO_6_:Eu^3+^ demonstrates a very low luminescence quenching behavior with its PL intensity still maintaining 81.6% at 423 K in comparison with that of the initial intensity at 303 K, as depicted in Fig. 8b. The excellent thermal stability is mainly attributed to its rigid network structure of double perovskite, which could adequately minimize the emission loss with enhancing temperature.^30,31^ Besides, in order to investigate the temperature dependence of luminescence in depth, the activated energy Δ*E* can be obtained by using the modified Arrhenius equation as follows^32–34^4
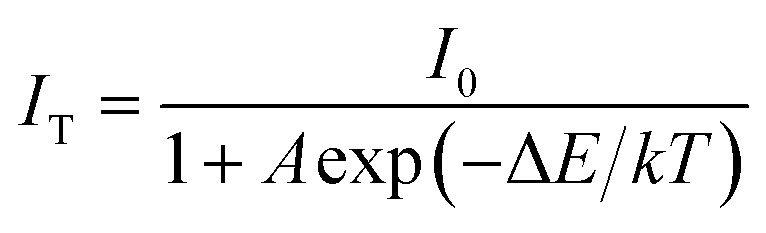
where *I*_T_ and *I*_0_ refer to the emission intensity of Ca_2_Y_0.3_Eu_0.7_SbO_6_ recorded at a different given temperature and the initial intensity at 303 K, respectively; *k* and *A* represent constants. The graph of ln(*I*_0_/*I*_T_ − 1) *versus* 1/*kT* is plotted and illustrated in Fig. 8c. It can be clearly seen that the experimental data is consistent with the fitting curve with the slope of −0.1838. Thus, Δ*E* is determined to be approximately 0.1838 eV, higher than some previous reports for red phosphors, such as Na_3_La_2_(PO_4_)_3_:0.02Eu^3+^ (0.0718 eV),^35^ or BaZrGe_3_O_9_:Eu^3+^ (0.175 eV).^23^ This relatively high Δ*E* demonstrates that the as-prepared phosphors have good thermal stability, further confirming that the red-emitting phosphor has potential application in LED devices. Besides, the energy difference between ^5^D_0_ and ^7^F_*J*_ of Eu^3+^ is illustrated in Fig. S2.† In this case, a very low luminescence quenching behavior mainly originates from the variations in temperature insufficient to match the energy level difference (∼1.414 eV).^36^ Therefore, the obtained Ca_2_YSbO_6_:Eu^3+^ phosphors exhibit excellent thermal stability.

**Fig. 9:**
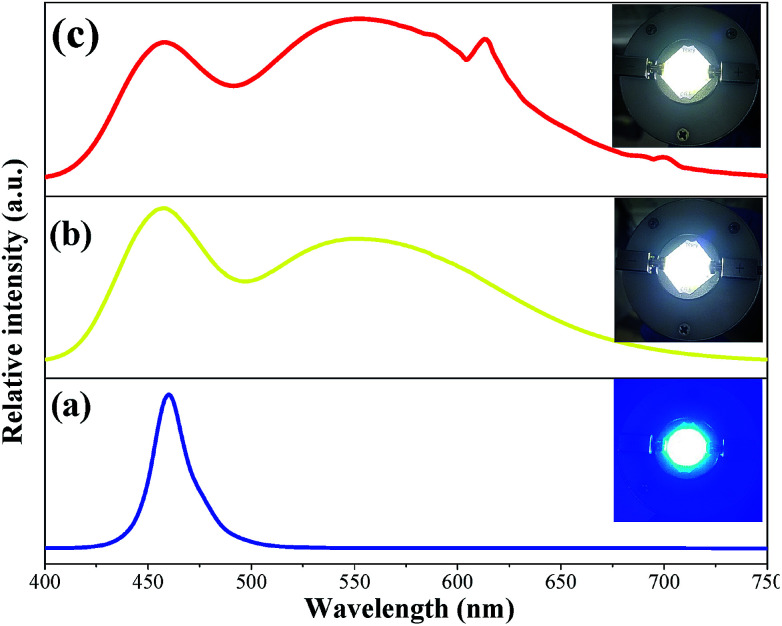
EL spectra of the LED device: (a) blue chip, (b) YAG:Ce^3+^ yellow phosphor and (c) Ca_2_YSbO_6_:Eu^3+^–YAG:Ce^3+^ mixture of phosphors combining with blue LED under 60 mA current excitation. Insets show digital photographs of the fabricated devices.

Additionally, the temperature-dependent luminescence decay curves of Ca_2_Y_0.3_Eu_0.7_SbO_6_ monitored at 612 nm were also measured, as illustrated in Fig. 9d. All the curves are well fitted to a single exponential and the decay behavior of Ca_2_Y_0.3_Eu_0.7_SbO_6_ weakly depends on the temperature. With the temperature increased to 523 K, the lifetime has slight variation, which further verifies that the as-prepared phosphor has excellent thermal stability.

### 3.3 Electroluminescent performance of the fabricated WLEDs

To further evaluate the potential applications of the Ca_2_YSbO_6_:Eu^3+^ phosphor, a prototype WLED device was fabricated by coupling a blend of the as-prepared red phosphor, the commercial YAG:Ce^3+^ yellow phosphor and transparent silicon resin on a 460 nm blue LED chip. The electro-luminescence (EL) spectra of the InGaN blue chip, the fabricated YAG:Ce^3+^-based and Ca_2_YSbO_6_:Eu^3+^–YAG:Ce^3+^ LEDs under 60 mA current excitation are depicted in Fig. 9. The corresponding EL spectrum in Fig. 9b contains two emission bands: the one at 460 nm belongs to the blue chip and the other at 550 nm is ascribed to the emission of YAG:Ce^3+^. Except the above emission bands, the peak located at 612 nm attributed to the emissions of Ca_2_YSbO_6_:Eu^3+^ can be observed in Fig. 9c. Bright cool white light is found (Fig. 9b, inset), and the CIE chromaticity coordinates, luminescent efficiency (LE), color rendering index (*R*_a_) as well as correlated color temperature (CCT) are measured to be (0.308, 0.341), 118.6 lm W^−1^, 75.4 and 6666 K, respectively. Thus, in order to improve the *R*_a_ and CCT of the LED, the as-prepared Ca_2_YSbO_6_:Eu^3+^ phosphor was added and the high performance warm WLEDs with a low CCT of 4720 K and high CRI of 82.6 can be achieved. Besides, the CIE diagram shifts to (0.353, 0.377) and the LE is 101.9 lm W^−1^. These results illustrate that the added Ca_2_YSbO_6_:Eu^3+^ red-emitting phosphor can effectively improve the photoelectric parameter of WLED devices.

## 4. Conclusions

In summary, highly efficient red-emitting Ca_2_Y_1−*x*_SbO_6_:*x*Eu^3+^ (0.1 ≤ *x* ≤ 1.0) phosphors with high concentration quenching and quantum efficiency have been successfully prepared by the traditional high-temperature solid state route. All the samples can be indexed to a pure monoclinic structure. The PLE and PL spectra demonstrate that the Ca_2_YSbO_6_:Eu^3+^ phosphors can be efficiently excited by NUV (393 nm) and blue light (464 nm), which match well with the UV and blue LED. Upon 464 nm excitation, a bright red emission located at 612 nm corresponding to the electric dipole transition ^5^D_0_–^7^F_2_ of Eu^3+^ can be observed. The optimal Eu^3+^ doping concentration reaches as high as 70 mol%, and the concentration quenching mechanism is attributed to the dipole–dipole interaction. Besides, the high quenching concentration mainly originates from different energy migrations between intralayer and interlayer luminescence centers in the Ca_2_YSbO_6_ lattice. Importantly, the IQE of Ca_2_Y_0.3_SbO_6_:0.7Eu^3+^ reaches up to 92.1%. Besides, the obtained phosphor demonstrates excellent thermal stability, where the emission intensity still maintains 81.6% at 423 K compared to that of the initial intensity at 303 K. This result is ascribed to its rigid network structure of double perovskite. Finally, a warm white light with CIE chromaticity coordinates of (0.353, 0.377), CCT of 4720 K and CRI of 82.6 can be achieved by efficiently fabricating Ca_2_YSbO_6_:Eu^3+^ phosphor in a 460 nm blue-InGaN chip together with the commercial Y_3_Al_5_O_12_:Ce^3+^ yellow phosphor. All of the results illustrate the promising prospect of Ca_2_YSbO_6_:Eu^3+^ as a potential red-emitting phosphor for application in blue/UV converted WLEDs.

## Conflicts of interest

There are no conflicts to declare.

## Supplementary Material

RA-009-C9RA03410B-s001
