# Supplementary material for: Highly efficient red-emitting Ca2YSbO6:Eu3+ double perovskite phosphors for warm WLEDs
Source: RSC Adv. 2019 Jul 3;9(36):20742–8. doi: 10.1039/c9ra03410b (PMC9066002; doi:10.1039/c9ra03410b)
Supplement: RA-009-C9RA03410B-s001 [file RA-009-C9RA03410B-s001.pdf]

Electronic Supplementary Material (ESI) for RSC Advances.
This journal is © The Royal Society of Chemistry 2019

Supporting information

Highly efficient red-emitting Ca_2YSbO_6:Eu^{3+} double perovskite phosphors for warm WLEDs

Meijiao Liu[a], Biao Shen[b], Keyuan Wang[b], Jiasong Zhong[b,*], Daqin Chen[c,*]

[a] Department of Chemistry, Zhejiang Sci-Tech University, Hangzhou 310018, China

[b] College of Materials and Environmental Engineering, Hangzhou Dianzi University, Hangzhou 310018, China

[c] College of Physics and Energy, Fujian Normal University, Fuzhou, 350117,P. R. China

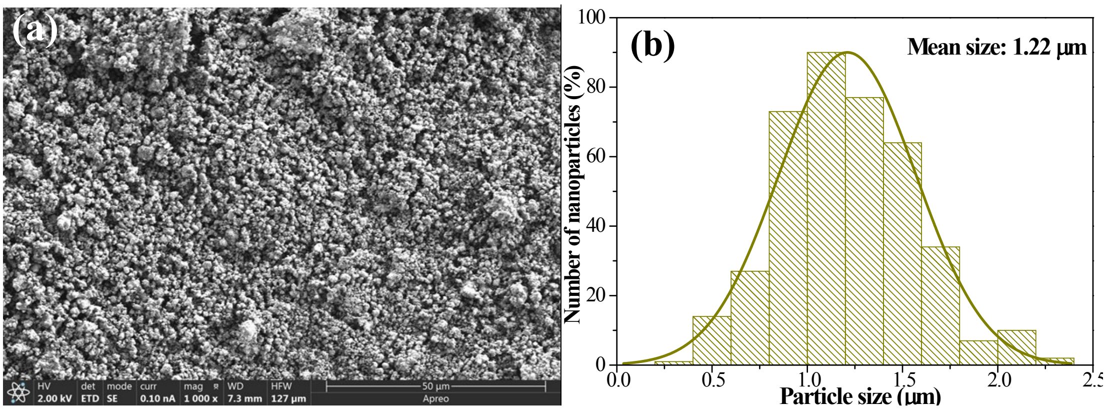

Fig. S1 (a) SEM image of $Ca_2Y_{0.3}SbO_6$:$0.7Eu^{3+}$ sample and (b) the corresponding size distribution image.

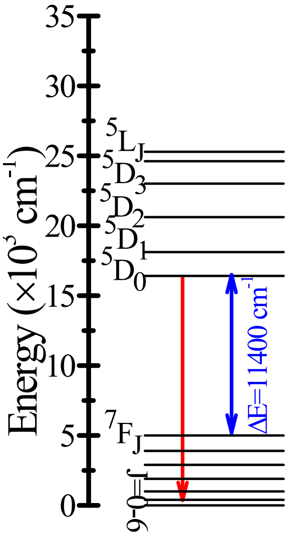

Fig. S2 The schematic energy level diagram of Eu^{3+}.

Table S1 Lattice parameters of $Ca_2YSbO_6:0.1Eu^{3+}$ sample

Formula	$Ca_2YSbO_6:0.1Eu^{3+}$
Crystal system	Monoclinic
Space group	$P2_1/n$ (14)
a (Å)	5.611
b (Å)	5.806
c(Å)	8.057
V (Å^3)	262.48
Chi^2	1.845
R_p	8.63%
R_{wp}	11.29%
R_e	6.12%
